# Editorial: Deciphering the inflammatory response in colorectal and ovarian cancers

**DOI:** 10.3389/fimmu.2026.1815051

**Published:** 2026-03-26

**Authors:** Sherin Bakhashab, Khurum Khan

**Affiliations:** 1 Department of Biochemistry, Faculty of Sciences, King Abdulaziz University, Jeddah, Saudi Arabia; 2 Institute of Genomic Medicine Sciences, King Abdulaziz University, Jeddah, Saudi Arabia; 3 Department of Gastrointestinal Oncology, University College London Hospitals NHS Foundation Trust, London, United Kingdom; 4 The London Clinic, London, United Kingdom; 5 HCA Healthcare UK, London, United Kingdom

**Keywords:** colorectal cancer, CtDNA, immune checkpoint inhibitors, inflammation, ovarian cancer, POLE mutations, tumor microenvironment

## Introduction

Inflammation is not merely a foundational hallmark of cancer; it is a programmable biological architecture that integrates tumour genomics, stromal conditioning, and systemic immune tone. Depending on context, inflammatory signaling may facilitate immune surveillance and tissue repair or, when chronic and dysregulated, drive genomic instability, epigenetic remodeling, angiogenesis, metabolic adaptation, immune evasion, and therapeutic resistance. In this sense, tumor-associated inflammation operates as an adaptive signaling interface between malignant cells and host immunity thus shaping not only tumor evolution but also therapeutic opportunity.

Colorectal cancer (CRC) and ovarian cancer (OC) represent biologically distinct yet immunologically convergent malignancies in which inflammatory signaling networks critically determine disease trajectory. CRC exemplifies inflammation-driven carcinogenesis, where chronic mucosal immune activation promotes mutational accumulation and microenvironmental remodeling. In contrast, OC often models inflammation-driven immune suppression, evolving within the cytokine-rich peritoneal cavity where persistent inflammatory gradients foster immune exclusion and therapeutic resistance. Together, these malignancies illuminate complementary dimensions of inflammatory biology, one rooted in mutagenic initiation, the other in suppressive progression.

The Research Topic *Deciphering the Inflammatory Response in Colorectal and Ovarian Cancers* assembles mechanistic, translational, and clinical investigations that collectively redefine how inflammatory circuits govern tumor–immune dynamics. Across intracellular kinase signaling, systemic immune indices, lymph node–based immune contexture, genomic proofreading defects, and checkpoint reprogramming, the contributions converge on a unifying principle: inflammatory architecture dictates therapeutic responsiveness. By integrating inflammatory profiling with genomic and immunologic insights, these studies move the field beyond descriptive associations toward precision immuno-oncology grounded in biological mechanism.

## Inflammatory signaling in colorectal cancer

CRC remains one of the most compelling paradigms of inflammation-driven tumorigenesis. Chronic intestinal inflammation enhances mutational burden through reactive oxygen species, activates NF-κB and STAT3 transcriptional programs, disrupts epithelial barrier integrity, and reshapes the microbiota–immune interface. These processes collectively generate a tumor-permissive microenvironment characterized by pro-angiogenic cytokine gradients, recruitment of immunosuppressive myeloid populations, and progressive T-cell dysfunction. In this setting, inflammatory signaling functions simultaneously as mutagen, scaffold, and immune modulator.

Li et al. identify and validate the VEGF/p38MAPK/HSP27 axis as a pro-tumor inflammatory circuit in CRC. This pathway mechanistically links angiogenic signaling with stress-activated kinase cascades, amplifying survival pathways and reinforcing inflammatory persistence. Activation of p38MAPK stabilizes HSP27, enhancing cellular resilience to oxidative stress and inflammatory cytokine exposure. Importantly, this axis operates at the intersection of vascular remodeling and immune modulation: VEGF signaling impairs dendritic cell maturation and promotes T-cell exclusion, embedding immune suppression within angiogenic expansion. Targeting this inflammatory node therefore offers dual therapeutic leverage — direct tumor inhibition and restoration of immune permissiveness.

Beyond tumor-intrinsic signaling, systemic inflammatory states exert measurable influence on CRC outcomes. Li et al. demonstrate that the pretreatment pan-immune-inflammation value (PIV), integrating neutrophil, platelet, monocyte, and lymphocyte counts, serves as an independent prognostic marker. Elevated PIV reflects expansion of immunosuppressive myeloid and platelet-mediated tumor-supportive populations alongside attenuated lymphocyte-driven cytotoxicity. As a composite metric, PIV operationalizes systemic immune tone and reinforces the concept that circulating indices are surrogates for microenvironmental dynamics. Incorporation of such biomarkers into therapeutic algorithms may refine risk stratification and identify patients requiring intensified or combinatorial strategies.

Cai et al. further extend this immune-informed perspective through the introduction of the log odds of negative lymph nodes/T stage ratio (LONT) in colon mucinous adenocarcinoma. By integrating depth of tumor invasion with the number of negative lymph nodes retrieved, LONT captures more than anatomical staging alone. The presence of negative lymph nodes may reflect both adequate surgical clearance and preserved regional immune surveillance. In this respect, LONT indirectly embeds elements of immune contexture within a pathological metric. Its improved prognostic discrimination compared with conventional tumor-node-metastasis (TNM) staging highlights the limitations of purely anatomical classification systems and supports the growing recognition that host–tumor immune interactions influence outcomes independently of stage.

Mahmood et al. provide complementary translational evidence that inflammatory and genomic dynamics are closely intertwined. Using circulating tumor DNA (ctDNA) to guide management of POLE-mutant gastrointestinal malignancies, the study demonstrates that defective DNA proofreading can generate profound sensitivity to immune checkpoint inhibition. POLE exonuclease domain mutations are associated with ultra-mutated phenotypes, increased neoantigen load, and prominent CD8^+^ T-cell infiltration. In three advanced cases, PD-1 blockade was associated with complete metabolic remission, sustained ctDNA clearance, and durable survival. Notably, one patient with microsatellite-stable gallbladder cancer harboring a non-exonuclease POLE variant also achieved a sustained response. These observations challenge the assumption that microsatellite stability uniformly predicts limited benefit from immunotherapy and suggest that specific genomic alterations may independently generate sufficient immunogenicity to overcome conventional resistance patterns. Importantly, serial ctDNA monitoring provided a dynamic correlate of therapeutic response, enabling real-time assessment of disease control and informing subsequent management decisions. This approach illustrates how genomic biomarkers can be integrated with inflammatory and immune parameters to refine treatment strategy. Rather than relying solely on static baseline markers, longitudinal ctDNA assessment offers a practical framework for adaptive immunotherapy, including consideration of treatment continuation, de-escalation, or surgical consolidation in selected cases.

Taken together, the CRC studies in this Research Topic span intracellular inflammatory signaling, systemic immune indices, pathological metrics reflecting immune context, and genomically encoded immunogenicity. They reinforce the concept that inflammation in CRC is neither uniform nor unidimensional. Instead, it operates across interconnected biological layers — from kinase activation to circulating immune composition — each with potential therapeutic relevance.

## Ovarian cancer: inflammatory immune suppression and therapeutic resistance

In contrast to CRC, where inflammation often contributes to mutational initiation and tumorigenesis, OC more commonly illustrates how persistent inflammatory gradients sustain immune suppression during disease progression. OC develops within the immunologically complex peritoneal cavity, where ascitic fluid maintains a cytokine-rich environment enriched in IL-6, TNF-α, VEGF, and TGF-β. These mediators promote macrophage polarization toward immunosuppressive phenotypes, impair dendritic cell function, and attenuate cytotoxic T-cell activation. Chronic peritoneal inflammation therefore contributes to immune exclusion and relative resistance to immunotherapy.

Asare-Werehene et al. redefine the functional scope of PD-L1 in OC. Beyond its established role as a membrane-bound immune checkpoint ligand, PD-L1 may localize to the nucleus, where it appears to regulate transcriptional programs associated with DNA repair and chemoresistance. This expanded functional perspective suggests that inflammatory checkpoint molecules can mediate both immune evasion and tumor-intrinsic survival pathways. Inflammatory cytokines such as IFN-γ induce PD-L1 expression, while nuclear PD-L1 has been implicated in resistance-associated signaling. These findings suggest that future therapeutic strategies may need to consider both immune-suppressive signaling and tumor cell–intrinsic adaptations.

Many ovarian tumors are characterized as immunologically “cold,” with enrichment of regulatory T cells, myeloid-derived suppressor cells, and alternatively activated macrophages within the tumor microenvironment. Reprogramming this suppressive milieu is likely to require selective modulation of inflammatory gradients rather than indiscriminate suppression. Approaches targeting macrophage polarization, chemokine networks, or metabolic constraints remain under active investigation and may, in selected contexts, enhance responsiveness to checkpoint blockade by restoring effector T-cell functionality.

## Translational integration and therapeutic opportunities

The mechanistic and biomarker insights presented in this Research Topic converge on a central translational theme: effective cancer therapy increasingly depends on integrating inflammatory biology with genomic understanding. In both CRC and OC, inflammation does not operate independently of tumor genetics; rather, genomic alterations shape inflammatory tone, and inflammatory context in turn influences therapeutic responsiveness. Precision oncology must therefore move beyond static molecular profiling toward integrated models that incorporate immune dynamics and longitudinal disease monitoring.

In CRC, targeting pro-tumor inflammatory signaling pathways such as VEGF/p38MAPK/HSP27 may enhance immunotherapy responsiveness by disrupting angiogenic immune exclusion and stress-adaptive survival programs. At the systemic level, composite inflammatory indices such as PIV provide accessible surrogates of host immune balance and may assist in identifying patients at higher risk of adverse outcomes. However, inflammatory profiling alone is unlikely to be sufficient. Its true clinical value emerges when interpreted alongside genomic drivers and dynamic biomarkers.

The POLE-mutant ctDNA-guided experience exemplifies this integrative approach. Defective DNA proofreading generates ultra-mutated phenotypes capable of eliciting robust antitumor immune responses, effectively encoding immunogenicity within the tumor genome itself. The use of serial ctDNA monitoring in this context extends genomic profiling from a baseline diagnostic tool to a real-time indicator of therapeutic effectiveness. Sustained ctDNA clearance, concordant with radiologic and metabolic response, illustrates how genomic-informed inflammatory responsiveness can be tracked longitudinally to guide continuation of immunotherapy, consideration of surgical consolidation, or adaptive treatment modification.

Importantly, these observations challenge the traditional dichotomy of microsatellite instability versus microsatellite stability as the principal determinant of checkpoint sensitivity. The POLE experience suggests that specific genomic alterations may independently generate sufficient neoantigen burden and immune activation to overcome conventional resistance frameworks. As such, ctDNA-informed identification of hypermutated or immunogenic subsets may refine patient selection beyond current categorical biomarkers.

In OC, where immune suppression predominates, similar integrative strategies may prove valuable. Understanding how genomic features interact with cytokine gradients, macrophage polarization, and checkpoint signaling could inform rational combination approaches designed to convert suppressive microenvironments into therapeutically responsive states.

Collectively, these studies support a shift toward biologically integrated trial design—where genomic alterations, inflammatory signatures, and dynamic biomarkers such as ctDNA are incorporated into patient stratification and adaptive treatment algorithms. Rather than viewing inflammation as a background phenomenon, it should be considered a modifiable and measurable determinant of therapeutic opportunity.

## Conclusion

The studies assembled in *Deciphering the Inflammatory Response in Colorectal and Ovarian Cancers* underscore a central insight: inflammation is not a passive hallmark of malignancy but a dynamic interface between tumor genomics and host immunity. When interrogated alongside genomic alterations and monitored through longitudinal biomarkers such as ctDNA, inflammatory context becomes clinically actionable rather than merely descriptive. Advancing outcomes in colorectal and ovarian cancer will depend on our ability to interpret and selectively reprogram these immune–genomic interactions with precision. In this regard, understanding inflammation is not adjunctive to cancer therapy—it is foundational to its future evolution.

